# The Hospital Nursing Supplement

**Published:** 1891-08-29

**Authors:** 


### "The Hospital" Nursing Mirror.

Being the Extra Nursing Supplement of "The Hospital" Newspaper.

Contributions for this Supplement should be addressed to the Editor, The Hospital, 140, Strand, London, W.C., and should have the word "Nursing" plainly written in left-hand top corner of the envelope.

### En Passant.

RARE COURAGE OF A NURSE.—Under this heading we drew attention on December 6th last to the rare courage displayed by Nurse Halkin, who came forward, and, in a long letter to the Committee, gave evidence in support of the patients' complaints against the food and arrangements at the Eastern Hospital. We ventured to predict that no laurels or thanks would be hers, and that her life would be a trying one for many days to come. As we expected Nurse Halkin found it impossible to remain at the Eastern Fever Hospital, but her action has now been fully justified by no less an authority than the Local Government Board itself. As will be seen from the Report of this Board dealing with the results of the special enquiry into the management of the Eastern Hospital, published in another column, the Government authorities declare that they have much doubt "whether having regard particularly to the occurrences in connection with the leaving of Nurse Halkin, the Matron has the judgment and discretion which are essential for a person filling the office she holds. While, however, they consider her conduct in this matter to be most reprehensible, they are reluctant to adopt the extreme course of requiring her resignation, and if the managers concur, in that case they will consent to the Matron continuing in her office for a period of six months on probation." Bravo, Nurse Halkin! You have certainly cause to rejoice that oblivion, thanks to your action, has not swallowed up the just complaints made against the mal-administration of the Eastern Hospital, and the thanks of the public and of all interested in the well-being of our hospitals are due to you for your public-spirited and brave act in a critical emergency. We do not know where Nurse Halkin is at present, but we hope that her employers will take note of the results of the Eastern Hospital enquiry, and that in due time a substantial reward will be hers, for she deserves compensation for the anxiety and loss which the courageous discharge of a public duty have brought upon her. All nurses may take courage from her example and rest assured that if they do right they will one day have their reward.

NURSING IN WORKHOUSES.—The adage "It never rains but it pours" is sometimes forcibly verified. Almost every week brings forth some fresh example of the inadequacy of the nursing in workhouse infirmaries. Some years ago Miss Twining took the matter up, and effected such a decided reformation in this department that the matter has been allowed to rest rather than progress with other branches of nursing. Nursing matters occupy an unusually large share of the public attention at present, and, as hospital nursing advances day by day to a higher state of efficiency, the eye-piece of public criticism is rapidly focussing the field of workhouse nursing. Last week we quoted the case of the nurse in the Preston Union, who was left for a short period in charge of eighty patients night and day. The shocking case of Charles Skelton, who died in the Strand Union, is now before us. Here the patient was placed in charge of a pauper inmate during the day and part of the night, although in a delirious condition. We are blaming the system, not the nurse, who was only acting according to custom in throwing so much responsibility on an unskilled person. The powers even of a nurse are limited, and the charge of probably nearly forty patients (which is no unusual number) is too great a tax upon anyone. A conference of the Local Government Administration was held lately at Gilsland, where an admirable paper on the subject was read by Mr. C. Hall, Medical Officer of the Carlisle Union. He proposed that an infirmary of 100 beds should have attached to it a superintendent nurse, a trained nurse, and probationers. It was also suggested that the infirmary should always be a separate department, not under the control of the Master or Matron. If these suggestions were carried out, union infirmaries would be placed on a proper basis.

KENT NURSING ASSOCIATION.—It is always a matter of regret when internal disturbances upset the working of a useful institution. The Lady Superintendent of the above Association has resigned, on account, it is stated, of the action taken by the Ladies' Committee in respect to the District Nurse, concerning whom unpleasant rumours were afloat. Her resignation has caused much regret amongst the nurses of the Association, one of whom writes: "Might I, as one amongst the numerous nurses, and on their behalf, state that never again could we hope to have at our head a lady who has so won our deferential love and esteem? It has been a source of great pleasure and comfort to us to hear from all classes without exception the almost universal sorrow at losing Miss Harbord. Particularly in homes of suffering and those desolated by the utmost poverty, will her presence be missed and her loss most severely felt." We trust that the petition, signed by twenty nurses, begging the Lady Superintendent to re-consider her decision, will have good effect, and that the Association and public may not lose such excellent services.

THE QUALITIES REQUISITE IN A NURSE.—An article on Home and District Nursing appears in a Belfast paper lately by a Belfast Physician, who says that a study of the requisites of a good nurse is essential to a girl who is thinking of taking up nursing as a profession. If she be fairly fitted for the work, but still wanting in some of the qualifications, a knowledge of what is required will be of great service to her; because during her training she can be cultivating and developing the qualities of mind and body in which she finds herself deficient. If this were not so, what would be the meaning of a nurse's "training"? Training is not merely instruction in the care of the sick. It involves a special drawing out or forming of the character for a special end. Her training is supposed to give her observation, patience, firmness, control of her feelings, thoughtfulness, and to increase her reasoning powers and judgment in as far as relates to the care of the sick. If it does not do all this, and much more, the training is a complete failure. It is really wonderful what can be done by cultivation when there is a strong wish for improvement. But though training does modify the character somewhat, it will not make the liar true, not the lazy industrious and painstaking. But, far short of such serious faults in a nurse, there are some very simple failings which entirely unfit certain women for nursing. For instance, there is the fussy nurse, the noisy nurse, the talkative nurse, the too quiet nurse, and the silly nurse, in spite of the best of training.

THE HITCHIN NURSING INSTITUTE.—A member of the above institution gives a gratifying account of the work done by the Hitchin nurses. He says: "Yesterday afternoon I thought I should like to see how the typhoid fever patients in Gascoigne's Yard were progressing (there are fourteen cases in the yard at this moment). I entered the yard. 'Have the nurses come yet?' I inquired. A dozen fingers pointed to a house. Miss Sawyer, the Lady Superintendent, kindly permitted me to accompany her on her inspection of the cases. She and her nurses visit the court twice a day regularly, and oftener when necessary. In the first house visited we found four children in various stages of illness. Now, what was the state of things as regards nursing in this house? The floors and stairs were scrupulously clean—kept so by the mother and elder sister of the patients, under the direction of the nurses. The children were lying on beds with clean bed linen; their faces, persons, and night-clothes were scrupulously clean and fresh, their hair neatly brushed. Order, sweetness, and neatness reigned throughout. Besides these fever cases the number of chronic cases under the care of Miss Sawyer and her assistants is very considerable. The people beg them to come in and visit ailing members of the family, and the doctors and district visitors apply to them freely for assistance.

### Asylum Articles.

### VI.—PICTURESQUE INCIDENTS.

There was peace in Nox Ward; the nurse was busy cutting out an apron, a patient was softly playing the Blue Danube waltz, and others were chatting and dozing. One patient certainly was walking restlessly up and down, and talking, talking, without ceasing; but then, this was her normal condition, she had never been known to be still or asleep during the many years she had been in the asylum.

Suddenly a "hooter" sounded, a long low note, and the patients roused themselves, and two or three quickly remarked, "the fire alarm!" and went to the window to look out, the charge nurse put down her scissors and called in the junior nurse who had been busy in the dormitory, then the charge nurse went swiftly into the corridors. Here there was haste, the nurses flying to their rooms had donned large waterproof over-alls and flannel hoods, and then seizing their crickels had run for the front entrance. Here stood the porter's wife, slate in hand, as each nurse or attendant came up she briefly remarked, "In the laundry," and noted the name of the nurse and the exact time to the second. The laundry attendants had swiftly cleared all the patients out of that block of the buildings, the clothes lay in the steaming tubs, the machinery went whirling on, but not a human creature was in the block within two and a half minutes from the time the alarm was given. On the roof, however, was the engineer; in the yard nurses were working a hand-hydrant, an ambulance stood close at hand, the fire-escape had been wheeled up by the male attendants, and the fire hose got to work. The medical officers, watch in hand, stood taking stock of all.

It was only a trial alarm to see if the attendants knew their fire drill properly; but it was all the more interesting and less painfully exciting than a real fire. These false alarms take place about four times yearly, and not the least useful part is that the patients get accustomed to the sound of the hooter, and move in an orderly manner out of any block from whence the alarm is given. Probably were there a real fire all danger to the patients would be over before it was known that it was not the ordinary drill alarm.

The saddest ward at Berrywood was, undoubtedly, the children's; little creatures from three to thirteen years of age, nearly all idiotic and subject to fits. It seems so terrible that the period of childhood should be blasted in this way, that from infancy even this curse should be borne and the world offer nothing worth having to these human creatures. But passing into the children's ward one morning I found the "musical nurse" playing a march, and the charge nurse, hands behind her back, marching round and round the room followed by a string of her charges. After the march came a song, accompanied by gestures, and then came kindergarten lessons. It was wonderful to see what these children could do through imitation, and how, in a few, the music could rouse them to what seemed almost a faint glimmer of intelligence. The kindergarten system has only been in vogue at Berrywood during the last few years, but the authorities are highly pleased with the success attained.

During the summer months, Berrywood dispenses with theatricals and concerts in favour of picnics and teas; but the sacred concert on Sunday evenings goes on all the year round. The patients who wish to attend gather in one of the largest wards, the musical nurse presides at the harmonium, and the medical officer takes the chair. First, a Moody and Sankey hymn, with a good chorus is sung, and then two of the nurses are called upon for a duet; then a patient, a little girl of eight, dressed all in white, stands up and sings a solo in a shrill childish voice. More hymns with choruses follow, and whenever the proceedings flag the medical officer joins in with vigour, and beats time with his hand, and generally keeps the concert going. It is not necessary to linger over these incidents of asylum life; quite enough has been said to show any would-be attendants what sort of existence they would have to lead if they enter the service of the mentally afflicted. But I should like to answer here the pertinent enquiry of a correspondent, who asks why, in the illustration, the nurse wore a large bunch of keys at her waist if the doors are always unlocked? Well, the doors are locked at night, and the doors of the acute ward, as before stated, are always locked, that necessitates one key. Then the authorities at Berrywood are by no means rash, and the knife-box of each ward is kept locked, poker is kept locked in the coal-box, the bread-cutting machine is kept locked. Also, each nurse wears a whistle, a key for the electric call, and a key for the fire alarm. The open-door system does not mean that dangerous weapons are left unguarded amidst irresponsible hands; but only that all appearance of restrain is hidden so far as possible, and that every effort is made to induce the patients to behave reasonably, and not to prevent them from behaving unreasonably. It has been proved that if you keep a patient in a ward where the windows have wire guards, directly that patient can get near an unguarded window, she smashes the glass. Keep her in a ward with unguarded windows, merely remonstrate, and have the windows mended if they are broken, and she will soon forget to smash them—in fact, will forget all about them, for the insane have short memories as a rule. In the same way, the patient in a room where the doors are locked and the windows barred, immediately wants to get out; she plans and thinks of nothing else. She cannot forget her desire to escape, the bars and the grating of the key press ever on her brain. There is but little hope that she will get better. But supposing the attack of mania in its wildest form is over, and exhausted and weak the patient is removed to the infirmary ward; here, lying in bed, though carefully watched, she is treated as one ill in body only, and when her strength returns she is moved to a pretty bright ward with unlocked doors and all sorts of privileges. She must be, indeed, a bad patient if she cannot appreciate the difference, and gradually get to understand that better behaviour means brighter surroundings, and that freedom is given to those who can control themselves.

It is not so many years since chains and whips were the approved medicines for cerebral disease; it will not be many years before trained attendants and brighter surroundings will be made obligatory in all asylums. All honour then, to those who are now leading the way.

### Christmas Competitions.

We want none of our readers to go away for their holidays without taking some piece of work for wet days, which, when finished, can be sent to us for our Christmas parcels. So heartily were the garments for adults which we distributed last year appreciated, that we want this year to have twice the number. To encourage all to help us in this way, and to add interest to the work, we offer the following prizes, which will be awarded in books or money as the winners choose: (1) For the best pair of socks knitted by a nurse, 5s.; (2) for the best pair of socks knitted by any Hospital reader, 5s.; (3) for the best made flannel shirt, 10s.; (4) for the best made woman's blouse, 10s.; (5) for the best made flannel petticoat, 10s.; (6) for the best made and best shaped dressing gown for an invalid cut out and made by a nurse, 20s. It will be seen that No. 1 and 6 are reserved for nurses only. With regard to No. 6 we specially hope for many entries, and if we secure them we propose to give more than one prize. Flannellette is cheap, and light, and warm, and would, therefore, form the best material for the dressing gown. In judging, four marks are given for workmanship, four for shape, and two for general appearance; therefore, it is not wise to spend time on elaborate trimmings. Long seams may be done by machine.

### Nursing in St. Kilda.

BY C. G. FURLEY.

ALTHOUGH it is only a few years since the island of St. Kilda became at all known to the public, it has already been described by a considerable number of summer visitors, who go there in the best and calmest weather, and even then are glad to quit its gloomy and storm-beaten shores. But the fugitive impressions of a tourist must necessarily be inaccurate and incomplete when compared with the experiences of a resident, and it is for this reason that the narrative of a nurse, who has spent a year on the island, is worth recounting.

A year ago the Rev. Mr. Fiddes, who had not long before accepted the pastorate of St. Kilda, came to Glasgow to look for a trained nurse willing to work in such a far-off and lonely place. He not only saw that his people suffered from lack of skilled attendance during the ordinary ills which flesh is heir to, but was especially shocked at the excessive infant mortality, owing to which the population of the island is getting smaller year by year. The cause of this is, however, one against which medicine and nursing fight in vain. Isolated from the rest of the world, the inhabitants of St. Kilda have inter-married again and again, until among the seventy souls its population numbers it would be difficult to find one who is not a relative, more or less near, of all the rest. For many generations these consanguineous marriages went on unchecked and unrebuked, and although the present clergyman denounces them even from the pulpit, it is not to be expected that the voice of one man will immediately outweigh the traditions of the past, and the prejudices of an ignorant and suspicious race. But the inevitable result has followed, and the inhabitants of St. Kilda are dwindling with every generation. Many children are born, but as a rule only the first-born lives, at most only two survive in each family. On the eighth day after their birth the infants are attacked with a disease resembling lock-jaw, which has hitherto proved incurable.

It can scarcely be said, however, that they are a sickly race; for only the hardy can survive the manifold insanitariness of the conditions under which they live. To be born in a "one-roomed house," heavy with the smell of sea birds' feathers and oil, superadded to a complication of the ordinary domestic odours, from which relief can be found only by inhaling the bleakest airs of the Atlantic, is a test of fitness for the battle of life which promptly ends any pretensions of the weak to the right of existence even for those who survive the perils of infancy; long life is not to be predicted, so dangerous are the modes by which they earn their bread. Hardly a cliff on the rock-bound coast but has a tale of death or mutilation attached to it. That must be when men, and women, too, for that matter, make a living by rifling sea-birds of their eggs and their young. For those who fall into the sea, either by slipping from a ledge of rock or through the breaking of the rope by which they dangle in mid-air, there is no further question of mortal help; in that turbulent sea it is a chance if their bodies are even seen again. But you will find on the island more than one person who, having fallen among the rocks and been rescued—not without some heroic risks being run by the rescuers—passes through the rest of life maimed and suffering, for want of some skilled hand to bandage wounds and bind up broken limbs. For such cases as these a trained nurse was invaluable. It was not easy, however, to find a person suitable for the task. Besides possessing the necessary medical and surgical knowledge, anyone who accepted the post must be able to speak Gælic, and, moreover, must make up her mind to remain for a year —communication with the mainland being impossible, except in the height of summer. In addition to this, she must be prepared to do without many of the comforts which most of us consider essential to life, and be ready to contrive medicaments and dressings out of the most unlikely materials.

Not without difficulty Mr. Fiddes secured a suitable person at a Glasgow nursing institution, and carried her off to his western isle. As the accident of being wrecked on the coast of Harris on the voyage did not shake her courage, he had reason to conclude that Nurse Chisenhall was not a person to be easily depressed; but, though she had some experience of nursing work in workhouse infirmaries, and in the poorer parts of more than one great town, St. Kilda contained for her some surprises in the way of dirt and discomfort. Nor did she find her efforts wholly appreciated at first. The natives are suspicious of strangers; the summer tourist has taught them this. They accept his gratuities, and, unfortunately, are beginning to rely on these as a source of income; but they object to his sketch-book, and his smiles, and his supercilious comments on their manners and customs. The nurse excited some prejudice at first by washing the cups before drinking when she was invited to tea in a friendly way, and otherwise showing a fastidiousness which was not in accord with the traditions of the island. She was even accused of an utter lack of religious feeling when she took away the ashes from the hearth on Sunday. The people are in some respects deeply religious, but their views are narrow and tinged with gloom; and it must be admitted that they do not carry the scrupulous particularity with which they keep the Sabbath into their observance of other commandments. But temperament and situation alike dispose them to sad and limited views of life. In the little town of sixteen houses, stranded on a horse shoe bay, at the end of the valley, which divides the lonely rock into two parts, there arises a small public opinion, which is more inquisitive about trifles than about weightier matters, and develops little proprieties rather than great moralities. The St. Kildans are more ready to see the evils of cooking on Sunday than the injury done by consanguineous marriage.

This gloomy temperament develops in the people diseases which are the last one associates with a hardy race whose occupations keep them much in the open air. Hysteria and melancholia are not unknown, and the nurse had to deal with one case of marked delusional insanity. A woman declared that she was blind, and could not be induced to admit that there was nothing wrong with her eyes until she was brought to Glasgow, when a doctor's tests and, perhaps, the sights of a great city compelled her to acknowledge that she saw perfectly well.

Even when care and kindness had won esteem, the nurse's post was not a pleasant one. The satisfaction with which she received a grateful pressure of the hand was modified by the knowledge that the said hand was impregnated with fulmar oil, with which the women lubricate their fingers when spinning yarn for tweeds and blankets. Spinning and weaving form the most profitable industry in the island, and is one which deserves encouragement. There is a loom in every house, and the tweed woven there from the undyed brown wool of the native sheep, that pasture on a neighbouring islet, could not fail to be popular if it were better known. At present only a small quantity is woven, and that passes through so many hands before it reaches the consumer, that very little of the price paid for it comes into the weavers' hands. It would be a great boon to the island if an agency were established, by which their goods could be conveyed to the public without being charged the numerous commissions that are now claimed by middlemen. Another thing, unfortunate for the natives, and probably not more fortunate for the proprietor of the island, is that rents are mostly paid in kind. Wool and feathers, taken for rent, may or may not produce the required sum when sold, while it is evident that when such things as cattle and sheep are taken for payment, the loss to the inhabitants is much greater than the price the animal will fetch at a market.

The valuation of the small brown native sheep is certainly moderate. The entire carcass of one may be bought for half-a-crown, but as the people are allowed to fill them only twice a year they do not bulk largely in their food supply. For the most part they subsist on fish, supplemented by the flesh of fulmars, puffins, and such strong-flavoured birds, not rendered more dainty by any skill in cooking. The nurse's modest culinary powers won hearts that could not appreciate her medical attainments. A cake, baked in an iron pot over the fire—there being no such thing as an oven in the island—was much talked of, as were other variations of the bannock of daily life. In some matters, however, her efforts achieved only contempt. She tried her hand at gardening, and though, as she regretfully admits, the onions were blasted by the frost, she was successful in rearing peas, beans, and potatoes. But she also managed to raise a few flowers, an achievement that won less admiration than she expected. "What was the use of flowers?" asked the St. Kildan's. "One could not eat them." They had no leisure for æsthetic cravings there.

And yet it is peculiar—and peculiarly Scottish—that in this narrow, sordid life, this hard struggle for even the barest subsistence, intellectual development should go on. Where in such an island in any other seas would one find a school at all—where, at least, would one find a school in which one of the pupils would attain such a proficiency in mathematics as would justify his being brought to the mainland to study further? One cannot help speculating on what the boy who has done this may become. The minister, who is schoolmaster, carpenter, and many other things as well, is not unreasonably proud of his prodigy, and wishes him to have opportunities for culture which St. Kilda cannot give. Mr. Fiddes is trying hard to civilise St. Kilda—no easy task when one reflects that for three-fourths of the year the island is cut off from the rest of the world by miles of stormy sea, and that for generations the inhabitants have been as far removed from modern influences as any Sandwich Islanders. His work, and that of the nurse, who may be regarded as his only ally, cannot be done in a day nor in a year, but the tears which the natives shed when their two benefactors set forth on a brief visit to the mainland surely show that it is well and successfully begun.

### The Princess of Wales' Fund for Mrs. Grimwood.

Nurse L. C. Carling wishes us to state that her name has been inaccurately given, first as Garling and then as Garbing. She has contributed 5s., and has received an acknowledgment in due course from Miss Knollys.

### Appointment.

Bedford Infirmary.—Miss Ada Smith has been appointed Matron of the Bedford General Infirmary. She received her training of three and a-half years at University College Hospital; was then Home Sister at the Hospital for Women, Soho Square, London, and for nearly two years served as Matron of the Bridgewater Infirmary, and lastly, was Home Sister of the Hospital for Sick Children, Great Ormond Street, London.

### Wants and Workers.

*Sister Agnes*, St. Mary's Cottage Hospital, Plaistow, would be very grateful for a pair of good disused crutches for a child of four with hip-joint disease.

*The Skipton District Nurse* would be glad if any kind friend would present a poor parish with a Bath chair for the use of wheeling out some invalid patients.

### The Princess of Wales and the Nurses.

Photographs are pouring in for the screen which the First and Second Thousand nurses have decided to offer for the acceptance of the Princess of Wales on her next birthday. As not more than 1,000 photographs can be placed on the screen those nurses who wish to show their appreciation of the Princess's kindness to them should send carte and postal order for 2s. immediately, to Screen, care of Miss Pritchard, The Lodge, Porchester Square, London, W., or they may be too late. Postal orders from the following Nurses are acknowledged: S. Teague, H. Harris, A. Sarson, I. Johnson, B. Westbuck, E. Lindsey, M. Smith, Melinda Smith, M. Payne, Aldwickle, G. Manning, A. Fairlie, E. Roberts, C. Libbis, Freeborn, I. Freeman, E. Wreford, A. Tame, M. Cassaidy, R. Piggott, L. Stubbs, F. Staunton, M. Taylor, M. Goff, Dannatt, F. Jarvis, E. Dennington, E. Peach, M. E. Smith, E. Gillingham, I. Thodey, S. Ransford, S. Graham, M. Thomas, A. Godwin, E. Marris, S. Arnold, H. Arnold, S. Baker, L. Dunn, M. Matthews, Jewell, M. J. Crocker, J. Blann, E. Keay, C. Vaughan, Perceval, Jackson, M. Williams, M. Churchill, A. Dossett, E. Myers, A. Taylor, M. Dickson, H. Hawkins, Jackson, Marsden, N. Blegner, Leeder, E. Cook, A. Garrett, Lealey, J. Landsborough, C. Rush, Leah, Pocock, F. C. Dugdale, A. E. Coker, J. Connell, R. A. Philips, E. J. Haddock, M. Smith, H. E. D. Coskin, F. Robinson, A. A. Nicholas, S. A. Hobbs, Annie E. Smith, F. Macdonald, J. L. Watts, E. M. Freeman, A. L. Long, S. Bardwell, E. Penny, A. E. Smith, A. Gurden, E. Connel, P. Lockyer, Arnold, Jordan, J. M. Davis, E. A. Fenton, A. Horan, F. Hunt, M. A. Woolley, M. E. Cardwell, A. Taylor, A. Brown, Cornford, A. Briggs, E. M. Dyne, S. Rees, S. A. Whitbread, A. J. Rowe, A. Spooner, A. Montague, A. Blackwell, M. E. Rootham, C. Buckle, A. M. Morton, E. Norman, Jacobs, Sister Margaret, Nurse Pyke, A. Stewart, J. Turner, E. Cook, F. Hillhouse, S. Stodgel, E. Blake, Rayner, E. Daines, Grey, Harré, E. Ayrton, Herley, E. Goss, S. Clarke, E. Thompson, S. Adams, E. Foster, E. Underhill, M. J. Thomas, S. Burton, E. Hoddinott, A. R. Lowe, M. H. Ormrod, E. E. Dudden, J. Wilcox, E. B. Quarry, M. Gates, C. Otterwell, C. E. Wilkins, A. Rowe, M. L. Roberts, E. Bradshaw, F. Williams, E. S. Wichelow, M. Cannon, E. Morley, L. C. Watts, A. Dormer, M. E. Barber, M. Miller, J. N. Dixon, E. Bird, E. H. Mullenger, E. Mapstone, H. M. Stevens, F. Green, J. Sherlock, A. Wakeling, M. M. Lloyd, S. Erplin, A. L. Bird, G. Guernsey, M. Wilson, J. Oram, E. Woolings, D. Jong, J. Pugh, H. Crellin, C. Mackay, M. Hudson, E. Pinder, H. Kendall, J. Barton, E. Randall, N. Solomon, E. Humphreys, C. Hawker, E. Cornwell, M. A. Winton, L. S. Leeder, E. M. Swalley, E. Pugh, Nelson, E. Elgar, A. Day, A. Bellamy, M. Bayes, J. Dearth, J. Nash, K. Pills, E. Sutcliffe, M. J. Pailey, L. Ridsdale, J. Davis, A. Peckham, M. Aldridge, J. Fleetwood, E. Liddle, A. Hickman, H. Osborne, K. Brown, M. Bailey, E. Edwards, F. Ready, C. Wellington, H. Fuller, M. Timpé, E. Pearson, W. Philips, F. Kirkwood, E. Cotton, H. Proschivitzky, Maunder, E. Digby, Satchell, K. McKenna, Murray, Winter, C. Phillips, and E. Jones.

Nurses who have not sent their photographs or postal orders for the screen are invited to do so immediately. Only 400 postal orders have been received, and about 150 more are promised. Unless a further 150 are received, it will not be possible to obtain a screen worthy of the Princess' acceptance.

Erratum:—Nurse C. Dalton was wrongly registered as Walton last week.

### Everybody's Opinion.

[*Correspondence on all subjects is invited, but we cannot in any way be responsible for the opinions expressed by our correspondents. No communications can be entertained if the name and address of the correspondent is not given, or unless one side of the paper only be written on.*]

### DRUNKEN NURSES.

"The Superintendent of a Nurses' Home" writes: "Last week I received into my home a nurse, who came to me from one of the large 'Nurses' Homes' in London. She came with a testimonial for ten years' good service in connection with that Home. I did not think there could be any danger in taking a nurse with such a character as that. What was my dismay when she got out of the omnibus to find that she was considerably the worse for drink! She would take nothing but a cup of tea, and insisted on going out again. In about half an-hour she returned, escorted by a crowd of boys, and so drunk that, even with their assistance, she fell several times as she came up the street. We got her to bed and sent for a medical man, who agreed with me that she must be sent home by the first train in the morning. Is it not a disgrace that such women should still be allowed to nurse the sick and dying? Please use this letter as you think best, omitting my name and address. It may be a warning to other institutions not to take a nurse only on the recommendation of a Secretary of a 'Nurses' Home.'"

### WANTED TWO HUNDRED AND FIFTY POUNDS.

A Correspondent calls our attention to a little spot in far-off Nova Scotia, where need of a hospital is felt very deeply. The inhabitants of Cow Bay, the little place in question, are too poor to provide the want, and it has been suggested that possibly some readers of The Hospital might render some assistance. We give the writer's own account: "Situated as we are on the sea coast, and in a mining district, where hundreds of British seamen and workmen come and go every year, much good could be done to body and soul if we possessed a small hospital. Last year a young man was sent on shore from an English steamer with typhoid fever. There was not a room to be procured; and, in spite of the poor fellow being able and willing to pay for immediate attention, and in spite of his critical condition, he had to be conveyed that night in an open waggon, thirty miles, over a bad road, to the nearest hospital. It is almost unnecessary for me to add that he died within a few days. During the present month, the widow of a late respected judge left Halifax by steamer for Sydney, accompanied by her son, fourteen years of age. It was discovered that he was developing diphtheria, which necessitated his being landed at Cow Bay. There was not a vacant room to be got, and for several hours the mother and son were made as comfortable as could be in the private office of the agent. At last a room was secured, and the boy transferred to it. A cold, draughty room, void of the commonest convenience, was the best that we could offer, and the boy died within forty-eight hours. I do not say that his death was caused directly by the surroundings, but no doubt they did their part."

### HURRY UP, NURSES!

"The Oldest Nurse Member of the Fund" writes: My dear fellow nurses and workers who have joined the Royal National Pension Fund for Nurses, I write these few words to tell you how anxious I am to see a very long list of names telling of photos, &c., received for "Screen" in next issue of The Hospital. I find that up to the 15th only 66 were sent. I should like to have counted over 500. Pray make haste, or "Screen" will be a failure. Should such a calamity befall, please stand firm not to have your money returned—beg that it may go on. A thing like this once set afoot must not really collapse. I, for one, would feel great shame, and I am sure many more would feel likewise. We well know that the sweet goodness of our loving and beloved President is greatly appreciated. We have had great honour and pleasure bestowed on us. Besides all this, such work and thought, both for our present and future good, from the founder, and from each gentleman and lady connected with the Fund. In fact, the great boon which this fund has conferred cannot be over estimated. In years to come its good will be felt more than to-day by thousands upon thousands, and those who are wise will join it. No more straits and anxious thoughts for future old age. That care taken off the mind. But I must not write any more now, only please hasten to send photos and postal orders for two shillings before the 5th September.

### A MATRON'S DUTIES IN REGARD TO NURSES' FOOD.

"A Superintendent" writes: Having employed a staff of eighty from different hospitals in the United Kingdom, I continually hear the complaint, and, I think, not without cause, of the want of variety in the nurses' diet, also the second-rate quality and indifferent cooking. To remedy this, I feel when a Matron is selected care should be taken to see that she has not only a certificate for nursing, but that she has a practical knowledge of the prices, the seasons, and the nourishment contained in the different kinds of food. Or where the hospital is large enough a housekeeper should be kept with these same qualifications. Secondly, the House Committee should visit and taste not less than twice a week (day unknown) the dinner provided for the nurses; for although they complain, they hardly ever like to in a formal way. I have been Matron over three years, and the first year I was questioned how it was I was able to have my expenses lower than my previous Matron and yet give the nurses the continual change I did? My reply was I had thoroughly learned in my own home, being the eldest of a large family. I put this to show that variety and quality need not entail extra expenses if rightly managed. For example, when I give the nurses an extra expensive dinner, such as veal and ham, the next day I give them tripe at threepence per lb. When salmon has been tenpence per lb. they have had a dinner of it. When there is cold meat and soup I give them fresh fruit instead of pudding; strawberries have been only threepence per lb. I make a change every day in the week for breakfast. And then the health and contentment of the nurses is kept.

### Notes and Queries.

### Queries.

(34) *Dunedin*, N.Z.—Could you tell me the climate, population, and cost of living in Dunedin, New Zealand? Is wearing material or clothes dear.—*A Constant Reader.*

(35) Can any of your readers tell me of a home where an old man would be received for a small sum? Is there such a place as a permanent home for the aged?—*Nurse Marjorie.*

(36)—*Medicus* would be glad to know where she can obtain india-rubber suitable for putting on shoe heels for wards to prevent noise.

(37)—Can any one tell me the price, and where I can obtain the Alpha Syringe, continuous flow, or advise any other with the same advantage?—*Monthly Nurse.*

(38) Can any one give me the name of an institution, whose terms are not too high, where a girl of weak intellect, but stubborn will, may be placed?—*A. L.*

### Answers.

(33) 1. If "An Exmouth Reader" will write to the Secretary, St. John Ambulance Association, St. John's Gate, Clerkenwell, E.C., full particulars would be sent of the best way to form and carry on an ambulance class under this old and valued Association. 2. The best way to set about an ambulance class in Exmouth is to get the printed directions from the St. John Ambulance Association, St. John's Gate, Clerkenwell, London, E.C. It is useless to try to start anything in Exmouth (or elsewhere) except on the prescribed lines. Mr. Domville, of Exeter, is a very good lecturer indeed.—*M. N. J. C.* 3. Miss E. B. Jackson, Gledfield, Ardgay, Rosshire, will be glad to correspond with "An Exmouth Reader."

(34) *Dunedin, New Zealand.*—"A Constant Reader" is informed that the population for 1890 is 46,443. The highest temperature in the shade is 93° C., and the lowest 26° C., the average being 57° C. The climate resembles Great Britain, but is more equable. Clothes and wearing material are higher than in England.

*Canadian Hospitals.*—"K. S." should refer to *Burdett's Hospital Annual* (140, Strand, W.C.), or to the Secretary to the High Commissioner for Canada (Victoria Chambers, 17, Victoria Street, S.W.). She might also write to Sister Frances, St. Luke's Home, Vancouver. Every letter should contain a statement of the training "K. S." has received, a copy of the certificate, and at least two testimonials of recent date.

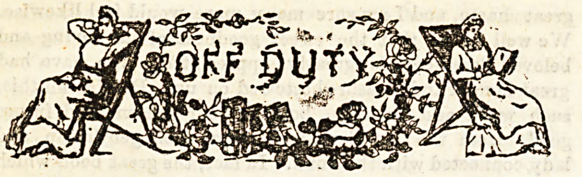

### On Pleasure Bent.

At Sandgate, when the sun shines and the waves lap the red sand gently—each little crest fringed by a sunbeam—and stretching back from the sea the broken mounds and hills rise sharply, their green summits crowned by the round Martello towers. Not yet has that for to the picturesque, the sanitarian comdemned Sandgate proper to fall at the feet of the builder, and the houses are for the most part huddled together in narrow and perplexing alleys, that seem, which-ever way you turn, to open upon the beach. From the tiny front garden of your lodging you look on a wide expanse of quiet sea, flecked by the white sails of the fishing-boats, and enlivened only by the skirls of the sea-birds; while the back window keeps you in touch with humanity by opening into an alley which affords boundless opportunities for studying the customs and manners of its inhabitants. They are mostly soldiers and fishermen's families, and appear to form a community in which everything is everybody's. Thus the woman at No. 1 shares the same wash-tub as the woman at at No. 3, and the babies seem to eat, sleep, and play in which-ever house they please. Under these circumstances it becomes an exciting problem as to whether the brown-eyed children with black hair belong to the brown-eyed mother on the top floor of No. 2, or the black-haired mother on the ground floor of No. 7. Also whether the delicate woman who is always tired is the wife of the tall soldier who lifts her heavy pails, or is she owned by the sturdy fisherman who comes round the corner and shouts to know if his tea is ready.

Visitors to Sandgate can rejoice in two climates. When the morning sun shines they can climb the heights behind and enjoy the wholesome breezes that are never absent, and when the evening shades commence they can descend and nestle in the shelter of the cliffs, where the air is still warm, and Lord Radnor has placed rustic seats for their accommodation. The inhabitants have not yet found out that lodgers are their natural enemies aud should be given the worst accommodation at the highest possible price, but take a tender interest in your welfare and unbosom themselves to you on family matters with a candour that is startling if not alarming. It is just as well, if you happen to be a nurse, to conceal your profession from these confiding folk, for it may be flattering, but it is not restful to have your opinion asked as to the causes and cures of the successive illnesses of a whole tribe of people. Just as affable are the fisher-lads, who row you about with a generous independence of time that often gives an hour-and-a-half for an hour's payment. There was one curly-haired lad, who became to us an intimate and valued friend. We were good sailors, and our enjoyment of a rough sea quite won his affections. When the sea was "choppy," and he had little chance of other customers, we got many an exhilarating sail in his fishing yawl, for which he would accept little payment. He would relate the most remarkable yarns and adventures, and once he imparted to us the recipe for his favourite dish of sea-gull boiled with an onion inside it; the onion, he said, took away the fishy taste. This knowledge will probably be useful when we are cast on a desert island. If we sat on the beach on a hot day our friend would rig up a shelter for our heads by means of a sail and four oars, and if the fishermen happen to catch any curious or little-known fish on their expeditions, it was always secured for our inspection.

On Sunday morning all fashionable Sandgate strolls up the hill to Shorncliffe Camp to see the soldiers march to church. This walk is not unattended with alarms; at unexpected intervals the sentries, who guard the approach to the camp, shout loudly, erecting their muskets, and bringing them down again with a terrific clatter that frightens the nervous, and sometimes causes them to take to their heels. There are stories told also of people who have flung themselves flat on the ground under the belief that the sentries were about to fire. I believe the sudden and war-like movement is called presenting arms, and as it always seemed to be executed just as we were passing we received it as an honour paid to our nurses' uniform. The soldiers' march to church is a most exciting sight and gives one a splendid idea of the shock the enemy would receive if he arrived between the two bands, each of which plays a different tune in a different key, with all its might and main. One brilliant regiment always attracted much attention, the men were of magnificent physique and appeared still more tall by reason of the curious erections on their helmets which were fastened to their shoulders by yellow cords, probably on account of the high winds in vogue at Shorncliffe. The church stands on the brow of the hill and the burial ground with its little white headstones slopes sharply down into the valley below. Away in the distance is Dungeness Point, and the little village of Hythe shows clearly in a far hollow, its square tower marking the place where the dry bones of unknown warriors are piled, bone upon bone, skull upon skull, in the curious crypts beneath. By following a path that leads around a mound you can look down upon the military hospital, which we were told was not open to inspection because the building was old and inconvenient. Query: Have the palatial buildings we wot of been putting little Shorncliffe to the blush? The convalescent patients may be seen strolling about the garden, their blue linen jackets contrasting prettily with the flecks of scarlet that dot the green hills and denote the presence of their healthier brethren. The grim Martello towers are peaceful dwellings now, and the soldiers cultivate cabbages and celery in the deep moats. Looking at these quaint little homes with their slender drawbridges, we undertood perfectly the determination of the hero of the song whose "name was Jim Boldyer, and he would be a Soldier;" evidently Jim wanted to get to Shorncliffe and live in a Martello tower.

### Amusements and Relaxation.

### SPECIAL NOTICE TO CORRESPONDENTS.

**Third Quarterly Word Competition commenced July 4th, 1891, ends September 26th, 1891.**

Competitors can enter for *all* quarterly competitions, but no competitor can take more than *one first prize* or *two* prizes of any kind during the year.

The word for dissection for this, the NINTH week of the quarter, being

"SQUADRON."

| Names. | Aug. 20th. | Totals. | Names. | Aug. 20th. | Totals. |
|---|---|---|---|---|---|
| Paignton | 4 | 241 | Christie | — | 44 |
| Psyche | 4 | 241 | Dulcamara | 4 | 234 |
| Hope | — | 47 | Nurse J. S. | 4 | 241 |
| Lightowlers | 4 | 253 | Qu'appelle | 4 | 245 |
| Wizard | 4 | 179 | E. M. S. | — | 68 |
| Wyameris | — | 46 | Jenny Wren | 4 | 224 |
| Dove | — | 46 | Carpe-diem | — | 65 |
| Punch | — | 181 | Grannie | — | 36 |
| Ivanhoe | 4 | 203 | Nurse G. P. | 4 | 137 |
| Tinie | — | 93 | Goodnight | — | — |
| Agamemnon | — | — | Gamp | — | — |
| Nurse Ellen | — | 86 | Charity | — | — |

**Notice to Correspondents.**

All letters referring to this page which do not arrive at **140, Strand, London, W.C.**, by the *first post on Thursdays*, and are not addressed PRIZE EDITOR, will in future be disqualified and disregarded.

